# The Effects of School-Based Maum Meditation Program on the Self-Esteem and School Adjustment in Primary School Students

**DOI:** 10.5539/gjhs.v5n4p14

**Published:** 2013-03-10

**Authors:** Yang Gyeong Yoo, In Soo Lee

**Affiliations:** 1 Department of Nursing, Kunsan National University, Kunsan, Korea; 2 Department of Emergency Health, Korea National University of Transportation, Zeungpyeong, Korea

**Keywords:** meditation, self-esteem, school adjustment, primary school students

## Abstract

Self-esteem and school adjustment of children in the lower grades of primary school, the beginning stage of school life, have a close relationship with development of personality, mental health and characters of children. Therefore, the present study aimed to verify the effect of school-based Maum Meditation program on children in the lower grades of primary school, as a personality education program. The result showed that the experimental group with application of Maum Meditation program had significant improvements in self-esteem and school adjustment, compared to the control group without the application. In conclusion, since the study provides significant evidence that the intervention of Maum Meditation program had positive effects on self-esteem and school adjustment of children in the early stage of primary school, it is suggested to actively employ Maum Meditation as a school-based meditation program for mental health promotion of children in the early school ages, the stage of formation of personalities and habits.

## 1. Introduction

### 1.1 Background

School, in conjunction with home, is the crucial environment in which children establish healthy socialization. In particular, the lower grades in the primary school make up the beginning stages of socialization training as a member of a society; therefore, adjustment to school life is a fundamental task to be resolved by children (Park, 2011; Yung & Choi, 2009). A matter of how well a child adapts to school life will carry on to how well he/she is able to adapt to school life in junior high school and high school, and will further influence his/her ability to adapt to the society as an adult. Therefore, the issue of a child's adjustment to school can be considered very important (Kim & Jang, 2007).

Modern society particularly has an environment where knowledge, values and behaviors quickly evolve. Assuming that a school is regarded as an institution that intentionally plays a crucial role in building up the ability of students to successfully adjust to school, such an issue as adjustment to school should be resolved (Jeong, 2005). Students, who successfully adjust to school life, have positive emotions, attitudes and motives about school, and thus, form good interpersonal relationships, perform better academically, and become able to contribute to a betterment of individuals and society by forming desirable behavioral characteristics (Seo, 2006).

As of April 2012 in South Korea, the number of students in primary, junior high and high schools has decreased; however, the rate of education discontinuation remains almost unchanged, in comparison to the previous year. The major reason for discontinuation of high school, which is not compulsory education in South Korea, is school maladjustment, comprising 43.9% of the total reasons (Korea Educational Development Institute, 2012). Many psychologists have claimed that self-esteem issue underlies the basis for such maladjustment (Joung, 2003), and reported that self-esteem or self-concept is one of the most notable factors of individual characteristics that influence children's school adjustment. According to Mack and Abon (1985), achievement of healthy self-esteem is one of the core tasks during the development phase for children and adolescents. In particular, achievement of self-esteem essentially relates to the existence of young children. Self-esteem is a significant factor that influences not only children's school lives but also their general life (Park & Lee, 2010), and it is acknowledged as a relatively persistent factor, thus, once it is developed in one's childhood, it is maintained for a rather long period of time through one's life. Therefore, formation of positive self-esteem in childhood is considered as a crucial task since it is essential to school life in secondary education and further influences his/her ability adjust to society as an adult (Seo, 2006; Choi & Jeon, 1993). That is, children who acknowledge and evaluate themselves as precious individuals have a high level of psychological resilience and a low level of sensitivity to external stimuli, and thus show high adaptability to the environment, meaning that they have excellent skills of school adaption (Kim & Lee, 2005). On the other hand, low self-esteem leads school maladjustment, violence or lapse, due to negative thoughts and passive behaviors, such as deliberate self-harm, sense of inferiority, worthlessness, depression, lack of motivation, self-dissatisfaction, and self-denial (Lee, 2011).

Accordingly, many schools are in search for an outstanding alternative to fulfill students’ unusual academic, social, emotional and behavioral demands (Wisner, Jones, & Gwin, 2010). As one of such alternatives, a variety of meditational programs are introduced at school sites (Wisner, Jones, & Gwin, 2010). Meditation is regarded as a new, promising practice and a state-of-the-art approach (Brown, 2007). In practice, meditation helps to improve students’ self-esteem (Benson et al., 1994) and stimulates emotional intelligence (Rosaen & Benn, 2006). Meditation integrated into school curriculums is likely to have positive influence on the school environment (Wisner, 2008).

On one hand, there has been increasing attention to Maum Meditation from many countries around the world, including South Korea (Lee, 2012). In addition, as attention to the educational values and significance of Maum Meditation highten, it has been employed not only as a program for classroom activities, club activities or creative experience activities at primary, junior high and high schools, but also as an official general education subject in some universities (Chung & Oh, 2009). Maum Meditation precisely defines the mind and scientifically suggests the method of cleansing the mind. It explains that the thoughts remembered from the life one has lived form the false mind, and are stored in the form of images. These memories are stored with emotions and continuously influence one's mind and behaviors. It holds the logic that one recovers the true mind, one's real mind, by throwing away the images, which are thrown away together with the emotions embedded in the memories (Woo, 2005). Cognitive counseling of the West approaches problem solving through changing the cognitive system because it places recognition and belief in causes of emotional occurrence. However, Maum Meditation helps to solve problems by directly eliminating the emotions embedded in the memories, which in turn eliminates dysfunctional thoughts of triggered conditions. Therefore, cognitive counseling and Maum Meditation ultimately hold both differences and similarities (An, 2006).

Works that verify the effects of meditation tend to increase in large scale, however, study participants in relation to meditation have mostly been adults, and studies have been rarely carried out with children and adolescents. Therefore, studies about school-based meditation programs are considered a prospective research field that is predicted grow significantly (Wisner, Jones, & Gwin, 2010).

Furthermore, if the purpose of education is to educate a person to have a happier and liberal life by easily adapting to his/her environment and adequately demonstrating one's ability, self-esteem will play a crucial role in the dignity of mankind and mental health. In addition, school adaption is profoundly considered as a significant issue for normal growth, development of children and maintenance of liberal life (Yoo, 2004; Jang, 2010). However, children in the lower grades of primary school, who need to adjust to unfamiliar environments and undergo a sudden change in their lives, have rarely been incorporated in the researches, although examination on their adjustment to school life has been in demand (Seo, 2006). Besides, efforts to develop programs to address improvement of their self-esteem and school adaption have been lacking.

In conclusion, self-esteem and adjustment to school life of children in the lower grades of primary school, a beginning stage of school life, have a substantial relationship with personality development, mental health and characteristics of children. Therefore, if the school-based Maum Meditation program is implemented to children as a personality education program, children are expected to have confident and positive attitudes toward school life in general as well as themselves, thereby imposing successful effects on instilling self-esteem and adapting to school. Based on this background, this study was carried out to verify the effects the school-based Maum Meditation program targeted to children in the lower grades of primary school have on self-esteem and adaption to school life in children.

### 1.2 Objectives

This study is projected to give assistance to children's school life and further to set a direction to establish a strategy for promoting mental health of primary school students, by verifying the effects of the ‘Maum Meditation program’ on self-esteem and school adjustment of primary school students. The specific objectives are as follows:

1) It is to verify whether self-esteem of the participants would improve after implementation of the Maum Meditation program.
2) It is to verify whether adaption to school would improve after implementation of the Maum Meditation program.

### 1.3 Hypotheses

Hypothesis 1. The experimental group who participates in the Maum Meditation program will mark higher in the self-esteem scores immediately and at 5 weeks after the program than will the comparison group.

Hypothesis 2. The experimental group who participates in the Maum Meditation program will mark higher in the school adjustment scores immediately and 5 weeks after the program than will the comparison group.

## 2. Method

### 2.1 Design

The study was a quasi-experimental study to understand and analyze the effects of the Maum Meditation program, by employing a nonequivalent control group pretest-posttest design (Table 1).

**Table 1:** Research design of this study

Groups	Pretest	Study Measure	Posttest	Further test
Experimental group	Ye_1_	X	Ye_2_	Ye_3_
Control group	Yc_1_		Yc_2_	Yc_3_

Ye_1_, Ye_2_, Ye_3_, Yc_1_, Yc_2_, Yc_3_: Self-esteem, school adjustment questionnaires

X: Maum Meditation program

### 2.2 Participants

A school selected for the study is N primary school located in a dense residential apartment area in K city, where middle and high schools are located right next to it. Thus, there have been some anxiety concerns from parents and children with regard to the children's school safety. For that reason, the school was appointed as a model school in which a variety of programs took places for safety education. Furthermore, the programs were aimed to satisfy demand for physical activities, to promote fitness and to prevent school violence. In addition, the school gates are kept locked and safety guardians were placed.

The parents of N school tend to have high educational qualifications and dual incomes, which means that they belong to above-average financial status of middle class. The parents, like most of the parents in major cities in South Korea, are keen to their children's academic performance, but relatively have less interest in the emotional or behavioral aspects of their children. Most of the children are involved in after-school programs for private tutoring regarding mathematics, English and essay writing. It is commonly observed that children are under severe stress due to fierce competition and academics; unfortunately, however, this kind of mental health has not gained much attention from both parents and teachers.

In this study, total sample size was determined by G*Power 3 program (Faul, Erdfelder, Lang, & Buchner, 2007). Since no previous research results were available about the effect size for the school-based meditational education program, the medium effect size f=0.25, level of significance α=0.05, power 1-β=0.85, and the repeated measures ANOVA between two groups with three measurements were set as a standard in this study. As a result, the minimum required sample size of this study was determined as 32. One class was allocated to the experimental group and control group, respectively, considering the failure rate due to early leave and absence since participants of this study were students in the lower grades of primary school.

For the study participants, the experimental group was composed of 26 students, a whole class of the third grade of this primary school, from March 2012 to July 2012. The randomly selected control group included 26 students, a whole class in the identical grade of the identical school, and was distant from the experimental group. Participants were provided information about the aims of the study, inclusion and exclusion criteria, and how data were to be managed. That is, participants were informed about the standard principles of protection of human subjects, such as the right to refuse, withdraw or discontinue participating in the study. All participants were provided written informed consent under principles of full disclosure, and a copy of the consent form was given to them. Moreover, they granted permission to have photos taken for the purpose of sharing in research reports. All the participants wished to participate in the study and their parents provided written consent.

The program began with 26 students in the experimental group and 26 students in the control group. However, a student from each group failed to complete the posttest due to family trip; thus, the attrition rate of the experimental group and control group was 3.8%, respectively. Therefore, a total of 50 students were the final participants of the study, 25 students in the experimental group and 25 students in the control group. None of the participants had any current medical or mental disorders that justified exclusion from the course, and none had previous experience of meditation. In addition, detailed demographic information was not collected.

### 2.3 Measures

#### 2.3.1 Self-Esteem

Self-esteem was measured using the self-esteem scale that was interpreted and modified by Jun (1999) from children's self-esteem inventory of Coopersmith (1967) and the self-esteem scale developed by Choi and Joen (1993). This inventory is composed of four subcategories; general self-esteem, social self-esteem, domestic self-esteem and academic self-esteem. The inventory is composed of 30 questions, using a scale of ‘Agree (3 points)’, ‘Not sure (2 points)’ and ‘Disagree (1 point)’. Inverse calculation was taken into place for negative questions. The total score was 90 points, but item mean score was presented, indicating the higher the score, the higher the self-esteem. The credibility of the data collected using the inventory had Cronbach's α of .854.

#### 2.3.2 School Adjustment

The scale to measure adaptability to school life was the behavior rating scale of Long and Henderson (1971) that was modified and reconstituted by Baeg (2002). Examination of school adjustment is composed of five subcategories; relationship with teachers, peer relationship, adjustment to school classes, compliance with school rules and satisfaction with school. The scale is composed of 30 questions, with answers of ‘Agree (3 points)’, ‘Not sure (2 points)’ and ‘Disagree (1 point)’. Inverse calculation was taken into place for negative questions. The total score was 90 points, but item mean score was presented, indicating the higher the score, the higher the school adjustment. The credibility of the data collected using the inventory had Cronbach's α of .859.

### 2.4 Procedure

#### 2.4.1 Development of an Experimental Intervention

1) Development of the school-based Maum Meditation program for mental health promotion of primary school students

In order to develop this program, two in-service teachers with experiences of implementing Maum Meditation at primary schools, a story expert and the researchers for this study composed the contents and methods of the first preliminary program after examining the materials and documents related to Maum Meditation Youths Camp for primary, junior high and high school students, which has been implemented since the winter of 1999. Next, the first preliminary program was modified and complemented by taking the advice from two professionals, professors from a school of education and degree of education. After this, second preliminary program, comprising a total of 15 sessions - 30 minutes per session - was composed. A program manual was written based on this preliminary program. Over two workshops at a conference of teachers who practiced Maum Meditation, feedback was collected from in-service teachers, and the final program was set in concrete after modification and complementation of the contents. For the method of mind subtraction, the method of level 1 was used among the eight levels of Maum Meditation (Table 2), and the contents of mind subtraction were formed from subtracting what has happened on the day and subtracting by topic. The program was composed of 30 sessions over a period of 15 weeks. The topic and the main content for each session are illustrated in Table 3. Each session was executed in a similar format, which is watching video clips or reading children's books to help children to understand the general concept of mind subtraction and implementation of mind subtraction, except for the sessions in the first 4 weeks when the explanations about the basic logic behind mind subtraction and the accompanying method took place.

**Table 2:** The eight levels of maum meditation

Level	Confirmation	Method
1	Level of knowing the universe is I	Throwing away remembered thoughts
2	Level of knowing there is no mind	Throwing away images of myself, images of my human relationships and myself
3	Level of knowing the universe is within me	Throwing away my body
4	Level of knowing the original soul and spirit	Throwing away my body and universe
5	Level of knowing the original soul and spirit and the world of the original soul and spirit	Throwing away my body and universe
6	Level of becoming the original soul and spirit	Myself goes into the black hole and dissolves and disappears and becomes the Universe
7	-	Destroying the entire the false world and myself completely
8	-	Level of being reborn as Truth by eliminating completely and doing the Action of Truth

**Table 3:** Main contents of school-based maum meditation program for mental health promotion of primary school students

Week	Theme	Main activities	Materials used
1	Orientation and Knowing the mind	· Knowing about the program (purpose and procedure)	Maum Meditation (presentation materials)
		· Knowing about the mind	
		· Knowing the logic of subtracting the mind and the method	White boards, board markers, board marker erasers, balloons
		· Knowing the true mind and the false mind	
2	Knowing the mind and Subtracting the mind	· Knowing the logic of subtracting the mind and the method	Maum Meditation (presentation materials)
		· Knowing the true mind and the false mind	
		· Recalling the memories	Seven Blind Mice (story book)
		· Subtracting what happened on the day	
3	Knowing the mind and Subtracting the mind	· Knowing the logic of subtracting the mind and the method	Maum Meditation (presentation materials)
		· Knowing the true mind and the false mind	
		· Subtracting what happened on the day	Powers of 10 (video clip)
4	Knowing the mind and Subtracting the mind	· Knowing the true mind and the false mind	Maum Meditation (presentation materials)
		· Subtracting the remembered thoughts in the chronological order	
		· Subtracting what happened on the day	Life is Short (video clip)
5	Subtracting the mind	· Subtracting the remembered thoughts of living self-centeredly in the chronological order	IKKODEMO HYAKKUNO RINGO (storybook)
		· Subtracting what happened on the day	Star Size Comparison (video clip)
6	Subtracting the mind	· Subtracting what happened on the day	Piggy Book (video clip)
		· Subtracting the thoughts remembered about family	Living as a Primary School Student in Korea, 2007 (video clip)
		· Subtracting the thoughts remembered about exams and study	
7	Subtracting the mind	· Subtracting what happened on the day	The Talmud: Selections (storybook)
		· Subtracting the thoughts remembered about likes and dislikes	Powers of 10 (video clip)
8	Subtracting the mind	· Subtracting what happened on the day	Wonhyo Buddhist Master and Haegolbagaji (storybook)
		· Subtracting the thoughts remembered about friends
		· Subtracting the thoughts remembered about difficulties and agonies	Empty Space (video clip)
9	Subtracting the mind	· Subtracting what happened on the day	Bakr, George (video clip)
		· Subtracting the thoughts remembered about cursing, violence and bullying	Water Knows the Answers (video clip)
10	Subtracting the mind	· Subtracting what happened on the day	Power of Words (video clip)
		· Subtracting the thoughts remembered about computer games and violent movie clips	
11	Subtracting the mind	· Subtracting what happened on the day	Maum Meditation (presentation materials)
		· Subtracting the thoughts remembered about fear, dread and scare	
12	Subtracting the mind	· Knowing the true mind and the false mind	Maum Meditation (presentation materials)
		· Subtracting what happened on the day	
		· Subtracting the thoughts remembered about competitive spirit and greed	Knowledge channel e Hamburger Connection
13	Subtracting the mind and Expanding the mind	· Knowing the true mind and the false mind	Maum Meditation (presentation materials)
		· Subtracting the remembered thoughts in the chronological order	
		· Subtracting what happened on the day	Let’s Live with Gratitude (presentation materials)
		· Reflect on what the self feels grateful for about the world and write them down	
14	Subtracting the mind and Expanding the mind	· Subtracting the thoughts remembered about what the self has done harm to friends, family, other people or things	Maum Meditation (presentation materials)
		· Subtracting what happened on the day	Do You Love Your Life? (video clip)
		· Confirming the real mind	
		· Carrying out appreciation for the world into action	
15	Expanding the mind	· Subtracting what happened on the day	What If the Earth is a Village for 100 People (video clip)
		· Confirming the real mind	
		· Carrying out appreciation for the world into action	
		· Sharing stories about their experience of Maum Meditation and resolutions	

2) Guides of the school-based Maum Meditation program for mental health promotion of primary school students

The guides of the program were in-service teachers, who completed the whole course of Maum Meditation and have been credentialed to guide the Maum Meditation program from the association of Maum Meditation. They also participated in the eight-hour-long workshop related to the implementation of the school-based Maum Meditation program.

#### 2.4.2 Development of an experiment intervention: Implementation of the school-based Maum Meditation program for mental health promotion of primary school students

The school-based Maum Meditation program for mental health promotion of primary school students was implemented from March 2012 to July 2012. It was implemented for 30 minutes each session, twice a week, for a total of 15 weeks. Training of mind subtraction was executed in the following ways, and changes were made to the details on a weekly basis.

1) In every session, children were helped to understand the logic of mind subtraction and to be motivated in relation to topics by employing children's books, video materials or presentation materials suitable for children. For instance, when they did mind subtraction activities with the topic of throwing away the thoughts in relation to cursing, violence and bullying, they watched a video book ‘Bark, George’ to understand that they naturally curse if they have cursing in their minds. Especially in week 1, in order to let them know about the real mind, they were asked to write 5 remembered thoughts they wanted to throw away and 5 materials in the world, and they played a ‘rock scissors paper’ game; when they won, they got to erase one, and when they lost, they got to erase two. Through this game, they could see that only the real mind remained in the end. In addition, in order to help them comprehend the logic of disappearance of false mind, they were asked to write the mind they wanted to throw away on balloons and popped them.
2) In order to let them acknowledge the contents of throwing away the mind, they were guided to find the false mind that they had in their minds and expressed it in writing or drawing.
3) Children were guided to close their eyes, recall the remembered thoughts and subtract them, according to the level 1 method.
4) Children were asked to write their feelings after subtracting the mind and present them to the class, therefore, the class could empathize with them. Especially in weeks 14 and 15, they were guided to confirm what the real mind was on their own.
5) Through expanding their mind after mind subtraction, they were guided to carry out appreciation to the world into action by cleaning a peer's place and tidying a peer's desk.

The above training was executed in the form of group program, and when the participants had difficulties due to their minds such as stress, individual counseling was supplemented.

### 2.5 Data Collection

#### 2.5.1 Preliminary Investigation

Preliminary investigation was carried out on 10 third-grade students on February 8, 2012, and the contexts were modified and complemented to be suitable for students in the lower grades of primary school.

#### 2.5.2 Pre-investigation

The researchers explained the purpose of the study and the means of data collection to the responsible class homeroom teachers. Homeroom teachers of the experimental group and control group received written consent regarding data collection from parents. Pre-investigation took place for the experimental group and control group in session 1 of week 1. Homeroom teachers of both groups directly read out the questionnaire to the participants. Some questions were reworded in easier expressions when the students could not understand the original questions. The students were asked to fill in the questionnaires by themselves.

#### 2.5.3 Post-investigation

Post-investigation was carried out twice; the first was executed immediately after completion of the fifteen-week program, and further investigation was executed after the summer vacation, on the same day for the experimental group and control group; it was 5 weeks after implementation of the first post-investigation. Data collection for this investigation was carried out using the same method by the same data collector as the pre-investigation.

### 2.6 Data Analysis

The data was analyzed using IBM SPSS Statistics 20.0 program. The sample size of the experimental and control groups was 25, respectively. Although the size was less than 30, it satisfied the test of normality. Therefore, a statistical analysis was conducted using parametric approach. To be more specific, homogeneity of the experimental group (25 students) who practiced Maum Meditation during classes and the control group (25 students) who did not practice Maum Meditation was analyzed by a specific demographic characteristic (gender) using the chi-square test (χ^2^-test). The mean (homogeneity) of pre-data about self-esteem and school adjustment of the experimental group and control group was analyzed using the t-test, and the mean comparison of post-data was analyzed using covariance analysis (ANCOVA). In order to compare the means of pre-data and further-data and of post-data and further-data about self-esteem and school adjustment of the experimental group, the paired t-test was computed. In order to examine the changes of patterns in self-esteem and school adjustment scores along with time, differences in self-esteem and school adjustment before, after and further after Maum Meditation between the experimental group and comparison group were analyzed using repeated measures analysis of variance (ANOVA). The differences were compared to examine whether there were aspects of change of data by different measuring times. As a result of examining the variables of self-esteem and school adjustment with the sphericity assumption, the self-esteem variable violated the sphericity assumption (Mauchly's sphericity test *p*=0.000); and thus, multivariate testing was used for analysis. On contrary, the school adjustment variable satisfied the sphericity assumption (Mauchly's sphericity test *p*=0.585); and thus, monovariate testing was employed. Furthermore, the Levene's test was used for analysis, which affirmed that the error variances of the dependent variables for each group were identical.

## 3. Results

### 3.1 Examination of Pre-Homogeneity between Participants

#### 3.1.1 Examination of Homogeneity about Demographic Characteristics of the Experimental Group and the Control Group

Both the experimental group and the control group were comprised of third-grade students of primary school. In order to examine the homogeneity regarding gender, the component ratio was observed. The gender ratio of the experimental group and comparison group was exactly the same, and thus it can be concluded that the two groups were identical in respect of gender (Table 4. χ^2^=0.000).

**Table 4:** Demographic characteristics of Maum Meditation group versus the control group (N=50)

Characteristics		Exp. (n=25)	Cont. (n=25)	Total (n=50)	χ^2^

		N(%)	N(%)	N(%)	
Gender	Male	12(50.0)	12(50.0)	24(100.0)	0.000
	Female	13(50.0)	13(50.0)	26(100.0)	

Exp.=Experimental group; Cont.=Control group

#### 3.1.2 Examination of Pre-Homogeneity about Dependent Variables of the Experimental Group and the Control Group

There was no significant difference in self-esteem and school adjustment between experimental and control groups before the program. The score of self-esteem before intervention was 2.32 (maximum value ~ minimum value: 2.73~1.77, range: 0.97) whereas mean for the control group was 2.28 (maximum value ~ minimum value: 2.63~1.73, range: 0.90). Also, the score of school adjustment before intervention was 2.37 (maximum value ~ minimum value: 2.67~1.67, range: 1.00) whereas means for the control group was 2.29 (maximum value ~ minimum value: 2.70~1.40, range: 1.30). In fact, the comparison of homogeneity about self-esteem and school adjustment of the experimental group and the control group before intervention was made through the t-test. No significant differences were statistically observed from self-esteem and school adjustment, within the significance level of 5%. That is, the two groups were identical in terms of each attribute (Table 5).

**Table 5:** Mean comparison of pre-data between the experimental group and control group (N=50)

Variables	Exp. (n=25)	Cont. (n=25)	t	*p*

	M±SD	M±SD		
Self-esteem	2.32±0.21	2.28±0.24	0.748	0.458
School adjustment	2.37±0.26	2.29±0.27	1.152	0.255

Exp.=Experimental group; Cont.=Control group

### 3.2 Examination of Differences in Self-Esteem and School Adjustment between the Participants

Table 6 shows the results of the mean comparison about variables of self-esteem and school adaption of the experimental group and control group after intervention through covariance analysis. Statistically significant difference was observed in both variables of self-esteem and school adjustment, thus it is evident that there was a difference in the means of post-data between the experimental group and control group. The effect size was larger than 0 for both variables, therefore, it is evident that the mean of the experimental group was larger than the mean of the comparison group. The effect size for self-esteem was 0.080, which did not contribute to the test results. However, it was 0.240 for school adjustment, which relatively contributed a large difference to the test results.

**Table 6:** Mean comparison of post-data between the experimental group and control group (N=50)

Variables	Exp. (n=25)			Cont. (n=25)			Effect size	ANCOVA (*p*)

	pretest M±SD	posttest M±SD	t-test (*p*)	pretest M±SD	posttest M±SD	t-test (*p*)		
Self-esteem	2.32±0.21	2.45±0.24	2.765 (0.011)	2.28±0.24	2.29±0.29	0.267 (0.791)	0.080	4.092 (0.049)
School adjustment	2.37±0.26	2.52±0.27	3.987 (0.001)	2.29±0.27	2.24±0.29	1.106 (0.280)	0.240	14.811 (0.000)

Exp.=Experimental group; Cont.=Control group

Table 7 shows the result of analysis of differences in the means of the data about self-esteem and school adjustment of the experimental group by time. For the self-esteem variable, a significant difference was statistically evident in both pre-at 5 weeks and post-at 5 weeks. However, for the school adaption variable, a significant difference was observed in pre-at 5 weeks, whereas no significant difference was observed in post-at 5 weeks.

**Table 7:** Mean comparison of data of the experimental group (N=25)

Variables	Pretest M±SD	Posttest M±SD	At 5 weeks M±SD	paired t-test (*p*)	

				pretest to At 5 weeks	posttest to At 5 weeks
Self-esteem	2.32±0.21	2.45±0.24	2.57±0.22	pre<FU (0.000)	post<FU (0.000)
School adjustment	2.37±0.26	2.52±0.27	2.57±0.25	pre<FU (0.000)	NS (0.255)

Exp.=Experimental group; Cont.=Control group; FU=Follow-up.

### 3.3 Hypothesis Test

#### 3.3.1 Hypothesis 1

The experimental group who participates in the Maum Meditation program will mark higher in the self-esteem scores immediately and at 5 weeks after the program than will the comparison group.

**Figure 1:**
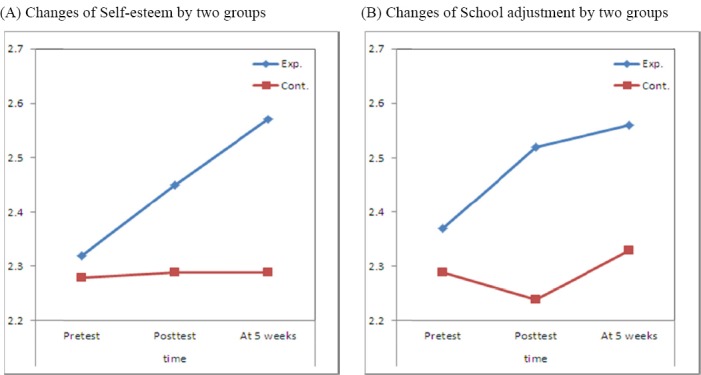
Changes of research variables by two groups Exp.=Experimental group; Cont.=Control group

For the experimental group, it increased from 2.32 (SD=0.21) of pre-test to 2.45 (SD=0.24) of post-test, and further to 2.57 (SD=0.22) at the follow-up test. For the comparison group, it was 2.28 (SD=0.24) at the pre-test, 2.29 (SD=0.29) at the post-test, and 2.29 (SD=0.29) at the follow-up test. It remained nearly unchanged. As a result of repeated measures analysis of variances analysis, a significantly conspicuous difference was observed statistically, between the experimental group and the control group (*F*=7.516, *p*=0.009). A significant difference was observed from along with time passage (*F*=5.396, *p*=0.008) and from the interaction between the group and the passage of time (*F*=4.798, *p*=0.013). Therefore, hypothesis 1 was supported. From this, it was evident that the aspects of change of self-esteem were different by groups along the passage of time (Table 8, Figure 1-A).

**Table 8:** Mean comparison of repeated measured data of the experimental group and control group (N=50)

Variables	Time	Exp. (n=25) M±SD	Cont. (n=25) M±SD	Between group	Within group	

				Group F(p)	Time F(p)	Time×Group F(p)
Self-esteem	pretest	2.32±0.21	2.28±0.24	7.516 (0.009)	5.396 (0.008)	4.798 (0.013)
	posttest	2.45±0.24	2.29±0.29			
	At 5 weeks	2.57±0.22	2.29±0.29			
School adjustment	pretest	2.37±0.26	2.29±0.27	7.738 (0.008)	8.010 (0.001)	5.694 (0.005)
	posttest	2.52±0.27	2.24±0.29			
	At 5 weeks	2.57±0.25	2.33±0.32			

Exp.=Experimental group; Cont.=Control group

#### 3.3.2 Hypothesis 2

The experimental group who participates in the Maum Meditation program will mark higher in the school adjustment scores immediately and 5 weeks after the program than will the comparison group.

For the experimental group, it increased from 2.37 (SD=0.26) at the pre-test to 2.52 (SD=0.27) and further to 2.57 (SD=0.25). For the comparison group, it slightly decreased from 2.29 (SD=0.27) at the pre-test to 2.24 (SD=0.29), and increased to 2.33 (SD=0.32). From the repeated measures analysis of variances, a significantly conspicuous difference was evident between the experimental group and the control group (*F*=7.738, *p*=0.008). Significantly conspicuous differences were statistically evident from along with time (*F*=8.010, *p*=0.001) and from the interaction between the group and the time passage (*F*=5.694, *p*=0.005). Therefore, hypothesis 2 was supported. This showed that the aspects of change of school adjustment were different by groups along the passage of time (Table 8, Figure 1-B).

## 4. Discussion

For many children, childhood is not a period without concerns and worries. In the social atmosphere of overflowing mammonism and instigating competition, children feel stressed and pressured as much as adults do. The minds of children are fully occupied by extremely bustling thoughts and emotions to the extent that children are unable to think and learn in their best way. Moreover, as they have difficulties with precisely expressing their problems, they can easily become a victim of frustration and rage of others. Because of this, they often experience negative emotions more deeply and intensely than adults (Fisher, 2006).

Many schools have been employing meditational techniques in order to address or prevent common emotional and psychological problems, such as apprehension and attention deficit disorder, which disrupt learning. Many parents, teachers and children participating in children's meditation programs have conviction in its benefits (Conis, 2005; cited in Fisher, 2006). Accordingly, the significance of this study is considered enormously profound in that it measured self-esteem and school adjustment of children in order to verify the effects of the school-based Maum Meditation program. In this study, the Maum Meditation program was implemented for 30 minutes each session, two sessions a week for a total of 15 weeks. Children's self-esteem in the experimental group was improved immediately after the program, compared to before experiencing the program. Also, the trend of continuous increase was shown after the five-week summer vacation. In the case of the control group, children's self-esteem remained almost unchanged immediately after the program or after the summer vacation, compared to before the implementation of the program.

It is assumed that our study results correspond to the study by Jung (2007) which reported that Mantra meditation had positive effects on the self-esteem of female academic high school students. A study by Yoo (1999) reported that meditation improved the self-esteem of high school students, irrespective of academic performance (cited in Joung, 2003). A study by Yang (2008) reported that junior high school students improved their self-esteem by growing their gratitude and realizing their values through gratitude meditation. A study by Cho (2006) reported that chakra mantra meditation was effective in changing the self-esteem of female junior high school students. Furthermore, the present study results are similar to the study by Kim (2006) which reported that meditation activities had positive effects on the self-esteem of children. A study by Kim (2003) reported that Buddhist meditation was effective in promoting self-esteem and developing sociality in children. A study by Benson et al. (1994) reported that eleventh-grade students who completed relaxation meditation by Benson (1975) as part of the academic curriculum showed significant improvement in self-esteem compared to the control group. A study by Beddoe and Murphy (2004) reported 75% of the participants had improvement in self-esteem upon completion of the Mindfulness meditation program. From the above studies which had implemented general meditation methods, it can be seen that negative emotions diminish as one becomes to be able to objectively observe one's negative recognition or emotions and that one newly attains positive self-esteem as one comes to see the self as he/she is (Joung, 2003).

Examining cases of previous researches with application of the Maum Meditation program that this study has attempted, it showed positive effects in improving self-esteem and self-efficacy as a result of applying the Maum Meditation program to third-grade children of primary school (Lee, 2009); it showed that self-esteem was significantly improved while depression, stress and apprehension were significantly diminished in college students after completion of Maum Meditation university students’ camp (Kim, 2009); and as a result of applying Maum Meditation youth camp to 476 primary, junior high and high school students, the students had their self-esteem improved compared to before experiencing the camp. This effect was particularly large on primary school students (Kim, 2012). Moreover, both qualitative and quantitative analyses of 10 students of a class out of 462 junior high and high school students who participated in Maum Meditation youth camp using

participation observation, in-depth interviews and questionnaires, it is acknowledged that the Maum Meditation program was effective in improving their self-esteem (Lee, 2011).

It is perceived that such results were obtained because when applying the Maum Meditation program of throwing away the thoughts remembered from the life lived until now, i.e. the false mind, it imposes the general effects of meditation, such as concentration, non-judgmental and accepting attitudes. Moreover, Maum Meditation is an active meditation method that enables one to naturally have a positive mind and change one's words and attitudes through improvement in self-esteem by helping one to directly throw away the false mind through getting rid of the negative mind stored in one's mind and then recovering one's true mind, that is, one's real mind (Lee, 2011; Jeong, 2006).

In this study, school adjustment of children was measured after applying Maum Meditation. The experimental group improved their school adjustment immediately after the program compared to before the program and showed a trend of continuous improvement at 5 weeks. However, in the case of the control group, school adjustment was slightly decreased immediately after the program compared to beforehand and showed a trend of slight improvement at 5 weeks, which was an insignificant change.

The concept of school adjustment in this study includes the relationship between teachers and students, relationship between peers, school classes, activities in classrooms, and participation in school events (Lee & Chung, 2009). School adjustment is broadly regarded as a construct in relation to academic performances, class attitudes and participation in the school environment (Mun, 2002; cited in Lee, 2003). Therefore, the concept of school adjustment is a complex one, rather than a single concept, that includes subcategories of the construct (Lee, 2003).

Examining the results of studies in relation to school-based meditation, a study evaluating the effects of transcendental meditation (TM) on stress reduction and behavioral management of African American students (Barnes, Bauza, & Treiber, 2003) reported that the experimental group that was intervened with TM twice a day, 15 minutes each, for a total of 4 months showed a reduction in the rate of absenteeism, violation of regulations and the number of days of suspension, compared to the control group that received health education for 15 minutes every day. As a result of the qualitative analysis after interviewing seventh-grade African American students who practiced TM twice every school day, 10 minutes each, for a year, Rosaen and Benn (2006) drew three important points of an increasing state of restful alertness, improvement in skills indicative of emotional intelligence and improvement in academic performance. They also reported that their academic performances improved with increasing emotional regulation capacity due to the state of restful alertness, and that improvement in their adaptability to environments was noticed during interviews and their patience and showed further enhanced generosity. In addition, in a study by Beauchemin, Hutchins & Patterson (2008) that evaluated the results after providing Mindfulness meditation (MM) to high school students diagnosed with learning disorder every school day for 5 to 10 minutes for 5 weeks, the students who completed the program showed improvement in social skills, a reduction in anxiety and improvement in academic performance. In a study by Wisner (2008) that evaluated the effects of MM on alternative high school students after providing the MM group program for all students, it illustrated the enhancements of interpersonal and intrapersonal strengths, family involvement, school functioning, and affective strengths. As a result of accompanying qualitative data analysis, Wisner reported that MM was helpful in increasing self-regulation and that meditation resulted in a calmer school community with a more positive school climate and less stressed, happier, more engaged students.

In addition, pilot studies about adolescents suggest that meditation enhances coping abilities and self-regulation and improves social relationship with peers, and thus promotes the relationship within the school community and improves the school climate (Wisner, Jones, & Gwin, 2010). TM guidance for adolescent students not only has educational achievements but also will promote physical and psychological health on a long-term basis (Elder et al., 2011). Moreover, school administrators and teachers reported that the student group who practiced TM at school positively influenced other non-mediating students and the school environment as a whole (Elder et al., 2011) Teachers also reported that meditation was helpful in creating a more-focused learning environment that is relatively happier and healthier (Fisher, 2005; cited in Fisher, 2006).

Although the above preceding studies in relation to meditation were not studies that directly evaluated the effects of meditation intervention on school adjustment, it is perceived that the results about school adjustment for this study corresponded to the above studies that had applied general meditational methods, when considering the concept of school adjustment as a complex concept.

Some comments are worth noting from the children's note books after Maum Meditation program in this study: ‘Before mind subtraction, when I saw friends not close to me, I felt uncomfortable and did not like them. But after subtraction, I came to know that the mind of discomfort was the false mind. If I have such false mind again, I will subtract the mind so that I do not get to hate them,’ and ‘after subtracting the pictures in my mind, the school life becomes better and fun.’ As evident from these comments, it is considered that as children recover their original self, the true mind, by subtracting the negative thoughts from the life they lived, i.e. the false mind, through Maum Meditation. This in turn naturally influences their school life in a positive way, and thus the adaptability to the school environment improves.

In particular, a notable point from the study results is that self-esteem of the experimental group was significantly further improved (*p*=0.000) at 5 weeks (2.57±0.22), compared to immediately after the program (2.45±0.24). Moreover, school adjustment also further improved at 5 weeks (2.57±0.25), compared to immediately after the program (2.52±0.27); however, the gap between the two was not substantial. This result is considered to be relevant to the secondary questionnaire results that 15 out of 25 students (60%) practiced Maum Meditation as they learned from school, at an average of 4.6 times a week, for an average of 10.9 minutes each time during vacation. This eventually implies the need to persistently practice mind subtraction. Thus, a more positive change is expected from children if Maum Meditation is persistently implemented at schools.

## 5. Conclusion

In conclusion, the study showed that the Maum Meditation intervention had positive influences on self-esteem and on school adjustment for the lower grade students of primary school despite of a small sample size. Therefore, we suggest employing Maum Meditation as a school-based meditation program for mental health promotion of children for formation of their personality and habits.

Based on the results of this study, we suggest the following. First, due to the small sample size, it was not possible to draw generalized conclusions. Therefore, future researches could implement this program using a larger sample size, i.e. a larger number of students. Second, this study relied entirely upon paper-and-pencil self-reports and did not include physiological measures of research variables. Therefore, future researches could include multidimensional outcome measures, which capture physiological or behavioral changes.
